# Beyond Chemotherapy: Present and Future Perspectives in the Treatment of Lymphoproliferative Disorders

**DOI:** 10.3390/biomedicines12050977

**Published:** 2024-04-29

**Authors:** Fulvio Massaro, Fabio Andreozzi, Tom Abrassart, Julie Castiaux, Hanne Massa, Ornella Rizzo, Marie Vercruyssen

**Affiliations:** Hematology Department, Institut Jules Bordet, Université Libre de Bruxelles (ULB), 1000 Brussels, Belgium; fabio.andreozzi@hubruxelles.be (F.A.); tom.abrassart@hubruxelles.be (T.A.); julie.castiaux@hubruxelles.be (J.C.); hanne.massa@hubruxelles.be (H.M.); ornella.rizzo@hubruxelles.be (O.R.); marie.vercruyssen@hubruxelles.be (M.V.)

**Keywords:** lymphoproliferative disorders, non-Hodgkin lymphoma (NHL), Hodgkin lymphoma (HL), immunotherapy, monoclonal antibodies, checkpoint inhibitors, bispecific antibodies, CAR-T cell

## Abstract

Over the past three decades, the treatment of lymphoproliferative disorders has undergone profound changes, notably due to the increasing availability of innovative therapies with the potential to redefine clinical management paradigms. A major impact is related to the development of monoclonal antibodies, checkpoint inhibitors, bispecific antibodies, and chimeric antigen receptor T (CAR-T) cell therapies. This review discusses the current landscape of clinical trials targeting various hematological malignancies, highlighting promising early-phase results and strategies to overcome resistance. Lymphoproliferative disorders encompass a range of conditions: while in Hodgkin lymphoma (HL) the goal is to reduce chemotherapy-related toxicity by integrating immunotherapy into the frontline setting, peripheral T cell lymphoma (PTCL) lacks effective targeted therapies. The review emphasizes a shifting therapeutic landscape towards precision medicine and treatment modalities that are less toxic yet more effective.

## 1. Introduction

**Figure 1:**
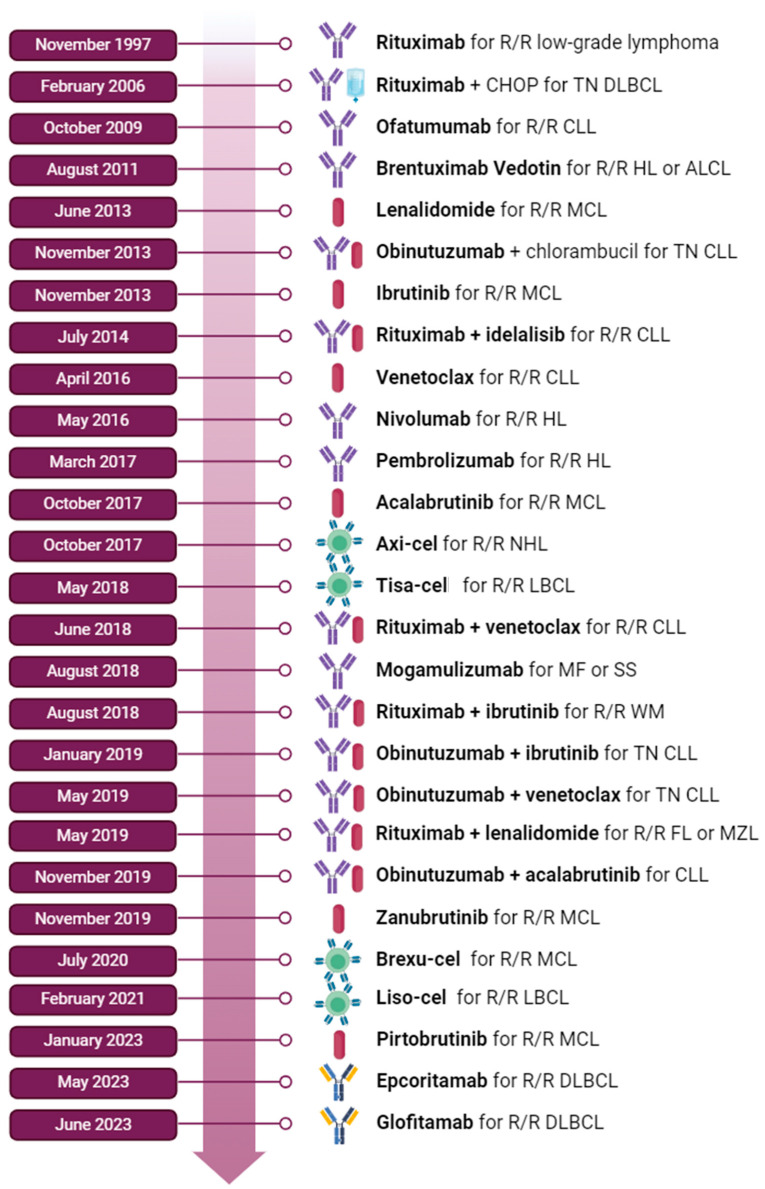
Developments in immunotherapy: FDA first approval for immunotherapies from 1997 to 2023. ALCL = anaplastic large cell lymphoma; CHOP = cyclophosphamide, doxorubicin, vincristine, prednisone; CLL = chronic lymphocytic leukemia; DLBCL = diffuse large B-cell lymphoma; FL = follicular lymphoma; HL = Hodgkin lymphoma; LBCL = large B-cell lymphoma; MCL = mantle cell lymphoma; MF = mycosis fungoides; MZL = marginal zone lymphoma; NHL = non-Hodgkin lymphoma; R/R = relapsed or refractory; SS = Sezary syndrome; TN = treatment naive; WM = Waldenström macroglobulinemia.

In the last 20 years, the treatment landscape for lymphoproliferative disorders has significantly evolved, particularly with the advent of immunotherapy. Since the introduction of rituximab, the first approved anti-cancer monoclonal antibody, in the late twentieth century, a wide array of biological therapies have been tested in clinical trials. These advances have paralleled a growing understanding of the disease biology and the pivotal role of the tumor microenvironment and the mechanisms of immune escape, leading to the development of new therapeutic strategies, including checkpoint inhibitors and chimeric antigen receptor T (CAR-T) cells. The enrichment of the therapeutic armamentarium has led to improved survival rates for patients with Hodgkin lymphoma (HL) and non-Hodgkin lymphoma (NHL), as illustrated in Figure 1 [1,2]. Contemporary research is also focused on reducing treatment-related toxicity. As patients live longer, it is crucial to prevent organ damage and preserve physical health. The use of chemotherapy is associated with a significant increase in long-term effects such as chronic fatigue, neurological and sexual impairment, infertility, and cardiovascular and metabolic disorders [3]. Although data on the frequency of late-onset toxicities associated with most of these new compounds are scarce, it is widely believed that their impact is generally less significant. For these reasons, clinical research for lymphoproliferative disorders is rapidly evolving, with numerous new trials exploring the potential of T-cell-based therapies in earlier disease phases, challenging the therapeutic paradigms that physicians have adhered to for decades. This narrative review aims to summarize the most promising treatments currently under investigation for HL and NHL. Due to the vast number of ongoing trials in this field, we have selected the most promising approaches with the aim of outlining the potential future scenario for managing lymphoproliferative disorders.

## 2. Early-Phase/Basket Trials

A wide variety of new molecules are currently under investigation in several phase I/II trials enrolling patients with different types of NHL. We report here a small selection of these studies, identified according to preliminary efficacy data and/or a particularly innovating and promising mechanism of action.

Golcadomide is an oral, Cereblon E3 ligase modulatory drug (CELMoDs), able to induce targeted degradation of the transcription factors Ikaros/Aiolos, which are crucial to B-cell malignancy development. In the phase I/II trial (NCT04884035) enrolling patients with relapsed/refractory (R/R) diffuse large B-cell lymphoma (DLBCL) or R/R follicular lymphoma (FL), golcadomide is proposed both in monotherapy and in combination with several partners. Efficacy data on 15 patients with DLBCL were recently presented: the overall response rate (ORR) was 50%, with 13% patients presenting a complete response (CR). Median duration of response (DoR) was 17.4 weeks. In the whole enrolled population of 35 patients, 17 (49%) had discontinued treatment, mostly due to progressive disease (*n* = 10, 29%) [4].

LP-168 is a next-generation BTKi which is highly selective for its target and has the dual ability to act both as a covalent BTK inhibitor (cBTKi) and non-covalent BTKi (ncBTKi) to overcome the mechanisms of resistance to common cBTKis caused by C481 mutation. In the phase I trial (NCT04993690), LP-168 monotherapy was employed to treat R/R B-NHL. Preliminary data on 60 patients reported an ORR of 65%: particularly, ORR in R/R mantle cell lymphoma (MCL) was 77% with a CR rate of 39%, non-germinal center (GCB) diffuse large B-cell lymphoma (DLBCL) had an ORR of 70.0% with a CR rate of 40.0% and marginal zone lymphoma (MZL) had an ORR of 73% with a CR rate of 9% [5].

Emavusertib (CA-4948) is an oral inhibitor of interleukin-1 receptor-associated kinase 4 (IRAK4), which is crucial for the toll-like receptor (TLR) and interleukin-1 receptor (IL-1R) signaling pathway in B-cell malignant proliferation. IRAK4 also acts by forming a complex with MYD88 adaptor protein leading to overactivation of nuclear factor-kappa B (NF-κB), promoting tumor growth. In the phase I trial (NCT03328078), emavusertib was given both in monotherapy and in combination with ibrutinib for patients presenting R/R B-NHL. The preliminary efficacy data of 16 evaluable patients in combination with ibrutinib showed five CRs (two for MCL and three for PCNSL) and one PR (one CLL). All three PCNSL patients with CRs were previously treated with BTK inhibitors [6].

HZ-H08905 is a first-class and potent CK1ε/PI3Kδ dual inhibitor, currently being investigated in a phase I study (CTR20213233) in B- and T-NHL. In the first reported analysis on 38 pts, HZ-H08905 in monotherapy had an ORR of 60%, including 17% CRs. Median DoR and progression-free survival (PFS) were not reached [7].

ZD8586 is a LYN/BTK dual inhibitor, which can arrest both BTK-dependent and BTK-independent BCR signaling pathways, giving the potential ability to overcome resistance to both cBTKi and ncBTKi. Currently, two phase I trials (TAI-SHAN1 and TAI-SHAN5) with oral administration of ZD8586 are enrolling patients with R/R B-NHL. In the cohort of 13 patients treated at doses ≥ 25 mg QD with at least one tumor assessment, the ORR was 69%, with responses observed across different histological subtypes, both indolent and aggressive NHL [8].

Zilovertamab vedotin is a humanized IgG1 monoclonal antibody directed against ROR1, associated with a proteolytically cleavable linker and the antimicrotubule agent monomethyl auristatin E. The transmembrane protein ROR1 has been found overexpressed in several hematologic malignancies. In the phase I waveLINE-001 study, zilovertamab vedotin showed promising efficacy after 14 months of follow-up, with an ORR of 29%, 53%, and 57% for patients with DLBCL, MCL, and Richter transformation (RT), respectively. In the phase II waveLINE-006 study, zilovertamab vedotin will be used both in monotherapy and in combination with the cBTKi nemtabrutinib [9].

The development of nanotechnology in oncology aims to increase drug delivery to tumor sites while limiting toxicity. AR160 is a novel 160 nm nano-immunoconjugate composed by nab-paclitaxel nanoparticles non-covalently coated with rituximab for targeted delivery into tumor cells expressing CD20. In the phase I study (NCI201601984), currently nine B-NHL patients have been enrolled. With a manageable toxicity profile mainly represented by hematological events, AR160 determined six partial responses (PR; 71%) and one CR (14%) [10]. 

A summary of the treatments discussed in this section is displayed in Table 1.

## 3. Indolent NHL

### 3.1. Follicular Lymphoma (FL)

In the setting of the first-line treatment of FL, the combination of rituximab and lenalidomide represents nowadays an alternative to chemoimmunotherapy [11]. This treatment presents a favorable safety profile and it is easy to manage: for this reason, it is widely proposed as a platform for combination with new agents in several clinical trials. 

A phase II study (NCT04404088), enrolling untreated FL needing a treatment as per the GELF criteria, evaluated the efficacy of acalabrutinib (100 mg twice a day, 28-day cycle, for a total of 13 cycles), rituximab (375 mg/m^2^ IV weekly during cycle 2, and on day 1 of cycle 3–13) and lenalidomide (20 mg daily on days 1–21, during cycle 2–13). In the 24 evaluable patients, the combination showed high activity, with an ORR of 100% and a CR rate of 92% and a 2-year PFS rate of 79%. Grade 3–4 toxicities were neutropenia (58%), transaminases elevation (17%), and infections (13%). Due to the rapid onset of CR (median time of 3 months), the protocol was modified to deliver only 6 months of treatment [12].

A class of drugs which is demonstrating significant activity in B-cell malignancies is represented by CD20xCD3 antibodies: recently, three of them (epcoritamab, glofitamab, and mosunetuzumab) have received FDA approval for B-cell NHL. They are active in both indolent and aggressive disorders and due to their excellent safety profile the number of clinical trials investigating their use in this clinical setting is rapidly increasing, with several studies also in first line. Mosunetuzumab is a bispecific antibody that has been approved by the FDA for the treatment of R/R FL after ≥ two prior lines of therapy, due to the efficacy data reported in a phase II trial in a high-risk population [13]. A phase II trial investigated the efficacy of subcutaneous (SC) mosunetuzumab in previously untreated FL patients (*n* = 43) meeting treatment indication according to the GELF criteria. Treatment lasted 8 cycles in pts who achieved a CR, and up to 17 in those with a PR. Twenty-six pts were evaluable for response: the ORR was 96% and the CR rate was 81%. All 22 CRs were seen at the first response assessment and were consistent in all high-risk subgroups (high FLIPI, bulky disease, grade 3A FL, SUVmax ≥ 13). The safety profile was manageable, with grade 3–4 adverse events (AEs) represented by neutropenia (10%). Cytokine release syndrome (CRS) occurred mostly after the first administration of mosenutuzumab and was G1 in 18 pts (90%) and G2 in 2 (10%) [14]. Mosunetuzumab is also under investigation in first-line FL treatment in combination with lenalidomide in a 12 month fixed-duration scheme (NCT04246086). Currently, 37 patients have been enrolled, with a promising ORR of 89% and a CR rate of 82% and a safety profile like that described for monotherapy [15]. 

In the R/R setting, other CD20xCD3 bispecific antibodies such as epcoritamab, glofitamab, and odronextamab were also highly effective, with ORRs of 81–90% and CR rates of 50–72% [16,17,18,19]. 

In the randomized phase II ROSEWOOD trial, the combination of zanubrutinib, a cBTKi, and obinutuzumab, an anti-CD20 monoclonal antibody, was compared to obinutuzumab as a single agent: the ORR was 69% and 46% (*p* = 0.001) and the CR rate was 39% and 19%, respectively. The 18-month DoR rate was 69% for the combination versus 42% for monotherapy arm and the median PFS was 28 months (ZO) versus 10 months, respectively (hazard ratio, 0.50; *p* < 0.001). The most common adverse events in the combination arm were neutropenia, thrombocytopenia, diarrhea, and fatigue [20].

A phase II trial (NCT04998669) investigated the activity of loncastuximab tesirine, an ADC containing an anti-CD19 monoclonal antibody, in combination with rituximab for R/R FL patients. Very preliminary results on 21 patients at a median follow-up of 4.8 months reported an ORR of 95% and a CR rate of 67% [21].

### 3.2. Mantle Cell Lymphoma (MCL)

Patients with TP53-mutated MCL have a poor prognosis with standard immunochemotherapies in frontline settings, representing today an unmet medical need, with a 2-year PFS rate of 20% [22]. In the phase II BOVen trial, patients with untreated TP53-mutated MCL were treated with a combination of zanubrutinib, venetoclax, and obinutuzumab for a minimum of 24 cycles, based on minimal residual disease (MRD) status. Among the 25 patients enrolled, the ORR and CR rate were 96% and 88%, respectively. At a median follow-up of 23 months, 2-year PFS rates and 2-year OS rates were 72% and 75%, respectively. Eight of the eleven evaluable patients for MRD at 24 months had a negative MRD and could discontinue treatment [23].

For R/R MCL patients, the cBTKi ibrutinib remains one of the most effective treatments, particularly in the first relapse, with a median PFS of approximately 2 years [24,25]. However, for patients becoming resistant to cBTKi, the outcome is dismal. 

The randomized phase III SYMPATICO trial compared the combination of ibrutinib and venetoclax (for 24 months, followed by ibrutinib alone) to ibrutinib as a single agent in patients with R/R MCL. Two hundred and forty-seven patients were enrolled and after a median follow-up of 51 months, the combination showed an advantage in the median PFS (32 months) compared to ibrutinib as a single agent (22 months; *p* = 0.005, HR: 0.65). Moreover, CR rate and time to next treatment were also in favor of the combination arm, with 54% versus 32% (*p* = 0.004) and NR versus 35 months (*p* = 0.01), respectively. Even if not statistically significant, probably due to a follow-up that was not sufficiently mature, mOS showed a tendency for improvement in the combination arm (44.9 versus 38.8 months, *p* = 0.35) [26].

Pirtobrutinib is a ncBTKi which is capable of inhibiting BTK activation despite the presence of mutations conferring a resistance to cBTKi. In the phase I/II BRUIN study, 152 R/R MCL patients were treated with oral pirtobrutinib monotherapy. A recently published analysis reported the efficacy data according to cBTKi exposure: naïve patients presented an ORR and 24-month DoR rate of 86% and 90% while for exposed patients the ORR and 24-month DoR rate were 49% and 39% [27].

A summary of the treatments discussed in this section is displayed in Table 2.

## 4. Chronic Lymphocytic Leukemia (CLL)

A few years ago, chemoimmunotherapy (CIT) was considered the gold standard for treating naive CLL patients with favorable cytogenetic and molecular characteristics. Currently, the fixed-duration treatment of obinutuzumab-venetoclax and ibrutinib-venetoclax are viable options for first-line treatment for CLL patients without TP53 aberrations [28,29,30,31]. On the other hand, several trials (E1912, FLAIR, ELEVATE TN, ILLUMINATE, SEQUOIA, and RESONATE-2) indicate that continuous treatment with cBTKi ibrutinib or acalabrutinib, with or without the association of obinutuzumab, are also good options [32,33,34,35,36,37]. However, in patients with TP53 aberrations, PFS continues to be shorter compared to patients without TP53 aberrations [28]. A continuous treatment with a cBTKi is currently considered the best option for treatment-naïve or R/R CLL patients with TP53 aberrations [38,39]. The efficacy of fixed-duration schemes compared to cBTKi treatment in first-line CLL is currently under investigation in the randomized trial CLL17.

Recently, some updates of important randomized phase III trials were presented. In the FLAIR trial, investigators compared a chemoimmunotherapy regimen with fludarabine, cyclophosphamide, and rituximab (FCR) to a combination of ibrutinib and venetoclax (I + V). Treatment duration for the I + V arm was tailored according to MRD levels, ranging from 2 to 6 years. Patients presenting a TP53 mutation or 17p deletion were excluded. A total of 523 patients were randomized to FCR (*n* = 263) and I + V (*n* = 260). The majority of I + V patients stopped the treatment after 2 (43%) and 3 (58%) years. At a median follow-up of 43.7 months, the 3-year PFS rate was 97% for I + V compared to 76% for FCR, with a hazard ratio (HR) of 0.13 (*p* < 0.0001), while the 3-year OS rate was 98% for I + V compared to 93% for FCR (HR of 0.31; *p* = 0.0029). Responses were consistent in all subgroups, with a particular advantage for unmutated IGHV (uIGHV) patients presenting 3-year PFS rates of 98% and 71% for I + V and FCR, respectively. The BM MRD negativity at any timepoint was 62% for I + V and 40% for FCR. Concerning safety, SAEs by organ class for FCR vs. I + V were: infections 18.8% for FCR and 22.2% for I + V; blood and lymphatic 31% and 5%; and cardiac 0.4% and 10.7% [33]. The I + V MRD-driven approach for CLL treatment seems a feasible and promising option but more mature data are needed to evaluate MRD persistence and PFS through the years after treatment discontinuation.

The GAIA trial compared three different fixed-duration venetoclax-based combinations (obinutuzumab + venetoclax, O + V; obinutuzumab + ibrutinib + venetoclax, O + I + V; rituximab + venetoclax, R + V) against chemoimmunotherapy (CIT) in fit, treatment-naïve CLL patients (*n* = 926) without 17p deletion or *TP53* mutation. A recent update at 50.7 months of follow-up was presented, reporting a median PFS that was significantly superior for O + V and O + I + V (not reached) compared to CIT (59.4 months) and to R + V (63.2 months). No significant PFS difference was reported between O + I + V and O + V except for uIGHV patients presenting an advantage with the triplet (HR 0.58; *p* = 0.025). No differences in OS were observed between the four treatment arms. Peripheral blood (PB) next-generation sequencing (NGS)-based MRD data were available for 816 pts at month 15: of these, 22.7% (52 pts, CIT), 23.6% (56 pts, R + V), 60.3% (138 pts, O + V) and 66.2% (153 pts, O + I + V) achieved uMRD < 10^−6^, which showed a significantly stronger correlation with PFS in all treatment arms if compared to MRD negativity according to conventional flow cytometry. Concerning safety, grade ≥ 3 infections were highest in O + I + V and CIT (CIT: 45 pts [20.8%], R + V: 27 [11.4%], O + V: 34 [14.9%], O + I + V: 51 [22.1%]) and cardiac disorders were most frequent in the triplet arm (CIT: 14 pts [6.5%], R + V: 19 [8.0%], O + V: 18 [7.9%], O + I + V: 41 [17.7%]) [40]. These data from the GAIA trial confirm the high efficacy of the fixed-duration venetoclax-based regimens and highlight the strong predictive role of NGS-based MRD if compared to flow cytometry.

Other ongoing studies are exploring different combinations of molecules, mainly second-generation cBTKi and BCL2 inhibitors (BCL2i), in the first line of treatment, in an effort to improve results achievable with the available agents. A phase III multicenter trial is currently ongoing, comparing acalabrutinib + venetoclax to obinutuzumab + venetoclax. In both arms, the duration of treatment is guided by clinical responses and minimal residual disease (MRD) status (NCT05057494). A phase III multicenter open-label trial (CLL16) will compare the addition of acalabrutinib to the combination of obinutuzumab and venetoclax in the first line for patients with high-risk CLL, defined as TP53 aberration or complex karyotype (NCT05197192). In a phase III open-label trial, a comparison is being conducted between the fixed duration of zanubrutinib + sonrotoclax and obinutuzumab + venetoclax (NCT06073821).

Concerning R/R CLL, according to PFS duration and the type of previously used agents, emerging evidence points out a potential role for the retreatment of relapsing patients with the same regimens used in first-line treatment. In a sub-study of the Murano trial, the retreatment strategy with venetoclax + rituximab was evaluated in patients who had previously undergone R/R treatment with the same regimen [41]. The median time since the last dose of venetoclax was 2.3 years. Most patients in this study exhibited at least one high-risk feature, such as unmutated IGHV, genomic complexity, and the presence of del(17p) and/or TP53 mutation. The ORR was 72%, with a median PFS of 23.3 months with a 56% probability of achieving uMRD at the end of treatment (EOT). There is an ongoing phase II study, the ReVenG trial, which is exploring the efficacy and safety of retreatment also with venetoclax + obinutuzumab [42]. Similarly, preliminary data from the CAPTIVATE trial support the use of retreatment with ibrutinib for patients who received a first-line fixed-duration ibrutinib + venetoclax combination: the ORR in 21 evaluable patients was 86%, mainly PR (81%). Eighteen patients have not received subsequent treatment while seven patients needed a subsequent line of therapy. Six patients have started retreatment with ibrutinib + venetoclax: best responses were CR in two cases, PR in three cases, and stable disease (SD) in one case [43].

The main clinical challenge and unmet need in R/R settings is represented by patients relapsing after a cBTKi and a BCL2i, for which currently there is no established standard treatment. Pirtobrutinib is a non-covalent BTKi (ncBTKi) which has shown promising efficacy in CLL patients previously treated with a cBTKi. In the recent update of the phase I/II BRUIN study, results on both the cohort of BCL2i-naïve (BCL2i-N; *n* = 154) and exposed (BCL2i-E; *n* = 128) patients were presented. The ORR was similar in the two groups, 83.1% for BCL2i-N and 79.7% for BCL2i-E patients, while the median DoR was 24.9 months for BCL2i-N and 14.8 months for BCL2i-E. With a median follow-up of 27.5 months, the median PFS was 19.4 months for all cBTKi pre-treated patients, 23.0 months for BCL2i-N, and 15.9 months for BCL2i-E. The 24-month OS rates were 73.2%, 83.1%, and 60.6% for all cBTKi pre-treated patients, BCL2i-N, and BCL2i-E, respectively. Concerning safety, the most frequent grade ≥ 3 AE was neutropenia (28.4%) while grade 3–4 hypertension (4.3%) and atrial fibrillation/flutter (1.8%) were infrequent [44]. Pirtobrutinib confirmed its efficacy in R/R CLL with a significant activity also in the more heavily pretreated population of BCL2i-E patients. 

In the phase Ib/II EPCORE CLL-1 trial, the safety and efficacy of epcoritamab for R/R CLL patients, either as monotherapy or in combination with venetoclax, is currently being evaluated. This trial enrolled a heavily pretreated cohort of cBTKi-exposed CLL patients: 65% had a TP53 aberration and 83% received also a BCL2-i. Recently, data from the expansion cohort of 23 patients were presented: the ORR was 82% with a CR rate of 33% and a sustained DoR rate, after 9 months of median follow-up, of 83% [45].

To address resistance to both covalent and non-covalent BTK inhibitors arising from BTK mutations, BTK degraders are currently under investigation [46]. Two ongoing phase I trials are assessing the safety of NX-2127 and BGB-16673 (NCT05006716) [47]. 

A summary of the treatments discussed in this section is displayed in Table 3.

## 5. Diffuse Large B-Cell Lymphoma (DLBCL) and Primary Mediastinal B-Cell Lymphoma (PMBCL)

DLBCL is the most common form of aggressive non-Hodgkin lymphoma [48]. Although progress in the development of new treatment regimens for the management of DLBCL has been relatively slow overall, recent developments in both the frontline and R/R settings have stirred optimism amongst clinicians in the field.

The phase III POLARIX trial evaluated the substitution of vincristine with polatuzumab vedotin, a CD79b-targeting antibody–drug conjugate in the Pola-R-CHP scheme. Adult patients (*n* = 879) with untreated intermediate–high risk (IPI 2–5) DLBCL were randomized to receive R-CHOP or Pola-R-CHP in a double-blind, placebo-controlled study [49]. Despite no overall survival (OS) benefit, the trial met its primary end-point of a statistically significant improvement in 2-year PFS rates of 6.5%, which led to its reimbursement in several countries. 

Recently, in the phase II Smart Start trial, Westin et al. demonstrated the feasibility of targeted chemotherapy-free frontline therapy with rituximab, lenalidomide, and ibrutinib (RLI), prior to chemotherapy in patients with newly diagnosed, non-germinal center B-cell-like DLBCL. The ORR after two cycles of RLI was 86.2%, and the CRR at the end of RLI-chemotherapy was 94.5%. With a median follow-up of 31 months, PFS and OS rates were 91.3% and 96.6% at 2 years, respectively [50]. Based on these data, the Smart Stop trial was conducted to evaluate if the number of chemo-immunotherapy cycles can be reduced or omitted after response to targeted therapy (lenalidomide, tafasitamab, rituximab, and acalabrutinib). All patients received LTRA for four cycles, followed by PET/CT scan. In cohort 1, patients received an additional six cycles of LTRA combined with a response-adapted number of cycles of CHOP therapy. After four cycles of LTRA, patients with CR received two cycles of CHOP, and all other patients received six cycles of CHOP. In the upcoming cohort 2, patients with CR after four cycles of LTRA are planned to receive no cycles of CHOP. The preplanned interim results from cohort 1 were presented at the last ASH meeting: after four cycles of LTRA, the ORR was 100% and the CRR was 64% [51]. 

Polatuzumab vedotin plus bendamustine and rituximab (pola-BR) received regulatory approvals for R/R DLBCL based on primary results from the randomized arms of the GO29365 study. The combination significantly improved metabolic CR rate (40% vs. 17.5%, *p* = 0.026), PFS (median 9.5 vs. 3.7 months, *p* < 0.001), and OS (median 12.4 vs. 4.7 months, *p* = 0.002) [52]. The results of the extension cohort (106 patients treated with pola-BR) of this clinical trial have recently been published and showed a metabolic CR rate of 39%, and a median PFS and OS of 6.6 and 12.5 months, respectively [53]. 

The combination of tafasitamab, a monoclonal anti-CD19 antibody, with lenalidomide has been approved by the FDA and EMA for patients with R/R DLBCL who are not candidates for ASCT following the good results obtained in the phase II L-MIND trial (*n* = 81), which featured high response rates (ORR, 60%, CR, 43%) [54]. A recent real-life study, published in 2023, which included patients with higher risk, was unable to reproduce the good results of the L-MIND trial. ORR and CR rate were 29% and 17%, respectively, and the median PFS and OS were 2 and 7 months, respectively [55].

Anti-CD20xCD3 bispecific antibodies epcoritamab and glofitamab received approval by the FDA in May and June of 2023 for the treatment of ≥3rd line R/R DLBCL. Epcoritamab was initially tested in phase 1/2 dose escalation in RR DLBCL. ORR was 63% with 39% CRs among evaluable patients receiving full doses [56]. A randomized phase III trial of epcoritamab vs. investigator’s choice chemotherapy in patients who did not respond to a previous ASCT or do not meet the criteria for ASCT is currently ongoing (NCT04628494). Its use in earlier lines, in combination, is also being investigating in the different EPCORE trials (NCT05578976, NCT05660967 or NCT05852717). Glofitamab approval was based on a pivotal phase II study (NCT03075696), including 154 patients with a median of three lines of treatment (a third post CAR-T cell therapy) demonstrating an ORR and CR rate of 52% and 39%, respectively [57]. Other bispecific antibodies are being examined, alone or in combination, including mosunetuzumab and odronextamab [58,59,60,61].

Even though most bispecific antibodies currently approved or under development for DLBCL target the CD20, different new approaches are being developed. GNC-038, the first-in-class octavalent, CD19xCD3x4-1BBxPD-L1 tetra-specific antibody, functions as a CD19-specific T cell engager by mediating direct antitumor activity, but also inhibiting T cells by PD-L1 based on its four binding sites (NCT05192486).

Multiple novel agents are currently under investigation for the treatment of R/R DLBCL. In the phase I first-in-human study in hematologic malignancies (waveLINE-001), the ROR1-targeting antibody–drug conjugate zilovertamab vedotin showed promising activity and manageable safety in R/R DLBC. ROR1 is an oncofetal protein pathologically expressed in hematologic malignancies including DLBCL. Early results of the phase II study in R/R DLBCL patients who are not candidates for ASCT and/or CAR-T showed that zilovertamab vedotin had clinically meaningful antitumor activity [62]. 

The antibody drug conjugate loncastuximab tesirine plus rituximab is being evaluated in the phase III LOTIS-5 study (NCT04384484); initial findings presented at the 2023 SOHO annual meeting showed promising initial efficacy and the study is expected to complete enrollment in 2024. Loncastuximab tesirine is also being combined with other anti-cancer agents in the phase Ib LOTIS-7 trial (NCT04970901). 

Despite sharing some biological and histological features with DLBCL, PMBCL is a distinct form of aggressive NHL that presents excellent outcomes with immunochemotherapy in first line. However, up to 30% of the patients will eventually relapse, representing a challenging population to treat, as they are often considered chemorefractory [63]. Copy number alterations (CNA) of 9p24.1 lead to increased expression of key genes including program death ligand-1 (PD-L1) and are frequently observed in PMBCL, making checkpoint inhibitors potential effective therapies. Indeed, the phase II KEYNOTE trial enrolled 53 R/R PMBCL patients with a median of three prior lines and reported an ORR of 41% with a CR rate of 20.8% [64]. The median DoR was not reached [64]. Those results led the FDA to approve pembrolizumab after two previous lines of treatment. 

Despite the disappointing results of brentuximab vedotin (BV) monotherapy in PMBCL, the phase II CheckMate 436 trial explored the combination therapy of BV and nivolumab in R/R PMBCL [65,66]. An ORR of 70% with 43% of CRs were observed, allowing new utility to BV in that setting [66].

A summary of the treatments discussed in this section is displayed in Table 4.

## 6. Primary Central Nervous System Lymphoma (PCNSL)

PCNSL is a rare type of diffuse large B-cell lymphoma that restrictively affects the brain, leptomeninges, spinal cord, and/or the eyes. Despite clear outcomes improvement in the last decades, 10–15% of patients would appear to be refractory to the first-line methotrexate-based treatment and up to 60% would eventually relapse [67,68] Currently, the R/R setting constitutes a medical need as no standard treatment has been established and the survival is dismal. In PCNSL, it has been shown that an L265P mutation on the *MYD88* gene constitutes an early mutational event in the development of the disease and is present with a prevalence as high as 71.7% [69,70]. BTK inhibition reduces NF-kB signaling and promotes cell apoptosis which makes BTKi a possible potent targeted therapy in the L265P *MYD88* mutated diseases, considering also the ability to cross the blood–brain barrier (BBB). A long-term analysis from a phase II study (NCT02542514) from French investigators was recently published, reporting encouraging data of ibrutinib as a single agent in the R/R setting: they showed an ORR of 59% with 19% CRs but disappointingly the median PFS was as short as 4.8 months and the median OS was only 20 months. Interestingly, patients with vitreoretinal involvement showed significantly better outcomes with a median PFS and OS of 69 months and 24 months, respectively [71]. Second-generation BTKi zanubrutinib is currently being investigated as monotherapy in R/R PCNSL (NCT05117814) after having proved its excellent ability to cross the BBB [72].

Other small molecules of interest are immunomodulatory agents (IMIDs), as they do cross the BBB and have a cytotoxic activity through NF-kB pathway inhibition but also a modulating activity on the microenvironment, stimulating natural killer (NK) and T cells against the tumor [73]. A phase II clinical trial evaluated the combination of lenalidomide and rituximab on 45 R/R PCNSL and showed an ORR of 67% but again a short PFS of 7.8 months [74]. Pomalidomide was also recently investigated in a phase I trial in combination with dexamethasone in 29 R/R PCNSL, resulting in an ORR of 48% with a modest median PFS of 5.9 months [75]. Several ongoing trials are currently exploring IMIDs in combination with different methotrexate-based regimens in first line (NCT04481815, NCT04446962, NCT04737889). Preliminary data of one of them were presented at the ASH meeting recently. The two-year PFS and OS rates were 62% and 67%, respectively, suggesting a potential improvement for this combination [76].

As immune checkpoint inhibitors have shown impressive results for CNS metastasis of melanoma, they were investigated in PCNSL. While PDL-1 and PD1 expression on tumor cells and their microenvironment cells is highly variable, clinical data are suggestive of high and prolonged efficacy: as an example, in a series of four patients with R/R PCNSL and one with CNS relapse of a testicular lymphoma, all patients responded to nivolumab, with one PR and four CRs. It is worth noting that three patients were progression-free at more than 12 months, making this approach highly attractive [77,78]. A combination trial with BTKi is currently recruiting PCNSL patients in first line (NCT04831658).

A summary of the treatments discussed in this section is displayed in Table 5.

## 7. Hodgkin Lymphoma (HL)

Historically, the management of HL has relied heavily on multi-agent chemotherapy regimens, coupled with radiation therapy for localized disease, resulting in high response rates and durable remissions. Despite this, their efficacy often comes at the cost of side effects and long-term complications, including the risk of secondary malignancies, cardiac complications, and infertility. Moreover, the small percentage of patients with R/R disease continue to face challenges in achieving sustained responses with current treatment regimens. This has spurred the exploration of innovative and targeted treatment modalities aimed at not only improving therapeutic outcomes but also mitigating the adverse effects associated with existing standards of care.

Recently, the use of BV associated with the frontline conventional ABVD regimen (with bleomycin omission) has shown improved outcomes compared to ABVD, especially in stage IV disease, multiple extranodal sites, and younger patients [79]. 

BV-AVD has also shown a high CR rate in limited-stage disease; however, the higher toxicity, mostly neuropathy and neutropenia, might not be appropriate in this low-risk disease category [80,81]. 

BV might also provide novel treatment options for older patients who historically have worse outcomes to combination therapy. The BREVITY trial included patients with stage IIB or bulky and advanced-stage disease, who were considered ineligible for chemotherapy, due to older age, frailty, or comorbidities. A BV monotherapy for a maximum of 16 cycles was given. Even though the treatment is well tolerated, the median PFS of 7.3 months and OS of 19.5 months are disappointing [82]. 

A different approach to improve tolerability is a sequential approach with chemotherapy. A recent phase II trial (NCT01476410) examined an induction by two cycles of BV followed by six cycles of AVD and a consolidation by four additional cycles of BV, in an older population. The results were encouraging, with a 2-year EFS, PFS, and OS rate of 80%, 84%, and 93%, respectively [83].

Due to tumor overexpression of PD-L1, the incorporation of PD-1 inhibitors, such as pembrolizumab and nivolumab, into the therapeutic arsenal for HL represents something which has already been established in an R/R setting and under development in first line [84].

As for BV, PD-1 inhibitors were also evaluated in combination with AVD. The NIVAHL trial studied the association of nivolumab and AVD (N-AVD), in both concomitant and sequential schemes. Both strategies proved safe and effective with a 12-month PFS rate of 98% and 100% for patients receiving sequential and concomitant treatment, respectively. Treatment discontinuation due to toxicity was necessary in 5 patients out of a total of 109 patients [85]. 

The unpublished results of the SWOG S1826 phase III study (*n* = 994) comparing the association of BV versus nivolumab in combination with AVD in advanced-stage disease were recently presented: at a median follow-up of 12.1 months, patients treated with N-AVD exhibited a higher PFS compared to those receiving BV-AVD (94% vs. 86%, respectively). N-AVD treatment was associated with increased incidence of neutropenia, but notably there was less use of granulocyte colony-stimulating factor (G-CSF) and no increased rate of infections. Conversely, the BV-AVD arm experienced more bone pain and neuropathy. Furthermore, a higher proportion of patients discontinued treatment in the BV-AVD arm (12% versus 8% in the nivolumab cohort). A longer follow-up will give more insights about the potential role as a new standard of treatment of N-AVD [86].

The association of BV and nivolumab has also been the object of a phase II trial (NCT02758717) hoping to develop a chemotherapy-free treatment in frontline settings for older patients and patients ineligible for chemotherapy due to comorbidities. The study unfortunately did not meet the prespecified criteria, but did demonstrate an activity as shown by the CR rate of 48%, and PR rate of 13%, giving an ORR of 61% [87].

The current standard of care in R/R settings is salvage chemotherapy, followed by ASCT for eligible patients, an approach which cures almost 50% of patients [88,89]. The addition of BV maintenance post-ASCT in high-risk patients (primary refractory disease, early relapse, or extranodal disease at relapse) has become the standard of care resulting in an improved PFS [90].

Multiple phase II trials have added BV to different salvage chemo regimens: in combination with bendamustine, ICE, IGEV, ESHAP, or DHAP, the CR rate ranges from 62% to 80% [91,92,93,94,95]. Studies with pembrolizumab show the most promising results, achieving complete remission rates before transplant of 87% in combination with ICE and 92% in combination with GVD with manageable toxicities, warranting further investigation in larger trials [96,97].

Chemo-free approaches combining different immunotherapies as salvage treatment showed encouraging results. The combination of nivolumab and BV determined complete metabolic remission (CMR) rates of 59% and 67% in two separate phase II studies (NCT02927769, NCT02572167) [98,99]. A small retrospective study evaluated the efficacy of pembrolizumab and BV as salvage treatment in 10 heavily pretreated patients, reporting an 80% rate of CMR [100].

For patients with subsequent R/R disease or those ineligible for ASCT, immunotherapy with BV or PD-1 inhibitors is currently the standard treatment, with discrete activity but low rates of durable CMR [101,102,103].

The association of CTLA4/KIR blockade with PD1 blockade has not improved outcomes in one phase Ib trial (NCT01592370) [104]. However, the combination of BV, nivolumab, and the anti-CTLA4 monoclonal antibody ipilimumab has shown promising results in another phase I trial (NCT01896999). Results of the phase II portion of the trial are underway [105]. 

Several new classes of drugs are currently under development for R/R HL.

The combination of lenalidomide with an mTOR inhibitor improves response rates in a phase II study (NCT01076543) with 20 heavily pretreated HL patients [106]. 

The antibody–drug conjugate camidanlumab combines an anti-CD25 monoclonal antibody with the drug pyrrolobenzodiazepine. The phase I trial (NCT02432235) has shown promising response rates in heavily pretreated HL patients [107,108]. 

The first-in-class CD30/CD16A-bispecific antibody AFM13 recruits NK cells to the tumor microenvironment. It has proven to be well-tolerated in phase I and II trials (NCT01221571, NCT02321592) with limited-efficacy AFM13 in R/R HL [109,110]. However, in combination with pembrolizumab, the objective response rate did increase in a phase Ib trial (NCT02665650) [111].

Tumor-associated antigen-specific T cells have been studied in a small cohort of patients with active R/R disease or in remission, with or without adjuvant nivolumab. The heterogeneity of this small group limits interpretation of efficacy, but did show disease control and a favorable safety profile (NCT022039303, NCT03843294) [112].

A summary of the treatments discussed in this section is displayed in Table 6.

## 8. Peripheral T-Cell Lymphoma, Not Otherwise Specified (PTCL-NOS)

Peripheral T-cell lymphomas derive from mature post-thymic T lymphocytes and mature NK cells; they account for around 10% of NHL [113]. The frontline therapy is based on anthracycline-containing regimens which are generally not curative, with CR rates of 44–64% [114]. Several attempts at adding a biological agent to chemotherapy, such as lenalidomide or romidepsin, failed to improve the outcome in this setting [115,116]. The Echelon-2 study, a randomized phase III study, compared BV and CHP versus CHOP in the frontline treatment of patients (*n* = 452) with CD30-positive mature T-cell lymphomas, showing an advantage from BV addition only for ALCL patients [117,118].

Consolidation with high-dose therapy and ASCT in first remission following frontline therapy showed conflicting data: D’Amore et al. published encouraging data from 160 patients with 50% OS and 44% PFS at 5 years corroborated by the Swedish register, but a retrospective study did not find superiority for the ASCT arm [119,120,121]. No significant difference was demonstrated for OS/EFS between autologous and allogeneic transplantation as part of first-line therapy in poor-risk peripheral T-NHL [122]. The ongoing phase III study TRANSCRIPT randomizes ASCT versus no consolidation after frontline chemotherapy for patients achieving at least a PR after the induction treatment (NCT05444712).

Prognosis of R/R PTCL-NOS is poor, with a 5-year OS rate reaching 10 to 50%, with a median PFS and OS rate, at less than 6 months [123]. Unfortunately, multiple trials trying to use new target therapies failed to demonstrate a significant efficacy in this setting: single-agent treatment with pralatrexate, romidepsine, belinostat, or alisertib was not associated with more than 6 months of median PFS [124,125,126,127,128]. Preliminary data on small cohorts of patients revealed new promising agents such as PI3K inhibitors duvelisib, linperlisib, and copanlisib (ORR 50%), the mtorc1 inhibitor everolimus (ORR 75%), proteasome inhibitors bortezomib and ixazomib (ORR 50%), and the EZH2 inhibitor valemetostat (ORR 55%) [129,130,131,132,133,134,135].

Probably, more interesting data will emerge from the combination of these target agents in doublets or triplets. Particularly, several combination regimens involving romidepsin have been tested (pralatrexate, duvelisib, and lenalidomide), showing encouraging ORR ranging from 50 to 71% in several small phase II studies [136,137,138,139,140]. The most promising association is represented by azacitidine and romidepsin: a phase II trial (NCT01998035) on 25 PTCL-NOS patients showed high rates of responses, especially for TFH PLTCL (ORR 80%, CRR 67%), and a retrospective real-world experience reported similar results [137,141].

Other new approaches with preliminary data have been recently presented. AZD4573 is a CDK9 inhibitor that downregulates MCL1, BFL1, and MYC overexpressed in PTCL-NOS. In the phase I study (NCT05140382), the preliminary analysis showed an ORR of 25% (all CR) in the efficacy-evaluable set of 12 patients [142].

HH2853, a novel dual inhibitor of EZH1 and 2, has demonstrated a clinical benefit in a phase Ib trial (NCT04390737) in R/R PTCL. In this study, 34 pts enrolled with different PTCL histology types, the ORR was 61%, including 21.4% CRs. The median DoR, PFS, and OS were not reached [143]. 

Golidocitinib is a JAK1 selective inhibitor which showed promising antitumor efficacy in patients with R/R PTCL (CTR20213318). In nodal subtypes, after a median follow-up time of 8.3 months, median PFS has not been reached, with 82.4% patients still responding [144]. 

A summary of the treatments discussed in this section is displayed in Table 7.

## 9. Cellular Therapy in Lymphoproliferative Disorders

Cellular therapy has witnessed remarkable progress in recent years, emerging as an effective and versatile approach for treating hematological diseases, with the most consolidate role in B-cell lymphoproliferative disease. While anti-CD19 CAR-T cells have demonstrated success in curing many cases of R/R B-NHL, a substantial proportion of patients (40–60%) exhibit non-responsiveness or relapse following treatment [145,146,147].

Multiple mechanisms are responsible for anti-CD19 CAR-T cell therapy failure, whose recognition is guiding clinical research in cellular therapy for lymphoma [148].

The production of commercialized autologous CAR-T cells can face challenges due to a reduced count of circulating lymphocytes and a weak cellular quality, due to disease factors and prior treatments. Additionally, the manufacturing process for autologous CAR-T cells needs several days, exposing the patient to the potential risk of disease progression before receiving the therapeutic infusion.

The constraints associated with autologous CAR-T cell therapy have instigated endeavors toward the development of allogeneic CAR-T cell products. The latter holds promise for enhanced cellular quality, obviating the necessity for lymphocyte apheresis, overcoming pre-infusion issues. Nonetheless, selection of allogeneic products should consider the risk CAR-T cell-mediated alloreactivity, which could precipitate graft-versus-host disease (GVHD) [149].

Strategies include selecting virus-directed specific T cells, genome editing, special subtype T cell selection, and utilizing umbilical cord blood as a T cell source [150,151]. Phase I studies on allogeneic CD19 CAR-T cells show comparable efficacy to autologous counterparts, pointing out the added advantage of rapid accessibility [152]. An ongoing investigation into haploidentical anti-CD19 CAR-T cells (NCT04887012) further exemplifies the commitment to advancing allogeneic therapies.

NK cells are emerging as an alternative to T cells in CAR-T therapy, offering advantages such as a lower risk of alloreactivity and different cytokine release profiles not responsible for cytokine release syndrome and neurotoxicity. Ongoing trials are evaluating allogeneic anti-CD19 CAR-NK cells (NCT05410041), as well as dual CAR-NK19/70 cells (NCT05842707).

Also, tumor-intrinsic factors contribute to current treatment failure, including the loss of CD19, a well-described mechanism responsible for relapse in about one-third of lymphoma patients [153].

Therefore, CAR-T cells targeting other antigens than CD19 are currently under investigation. CD22, with its prevalence in over 90% of B-cell malignancies, has shown promise in single-CD22-CAR-T cell trials, achieving favorable response rates [154]. Additionally, CD20, CD70, and CD79b are being investigated in phase I trials (NCT05948033, NCT05773040), offering potential alternatives to augment the therapeutic arsenal [155].

To circumvent susceptibility to antigen loss of CAR-T cell products directed against a single antigen, multiple target therapies are being actively explored. 

Strategies include using two separate CAR-T cell products, a single product expressing two CARs, or a single CAR binding two different antigens (tandem CARs). Early results from phase I trials on dual codominantly expressed anti-CD19/CD22 CAR-T cells showed potent and prolonged anti-lymphoma effects, highlighting the need for continued exploration [156,157,158].

Tumor microenvironments can also be implicated in CAR-T cell failure: in a recent publication, a more hypoxic and immune-suppressive microenvironment, comprehending myeloid-associated immune-suppressive and TGFβ-activated stromal genes, was associated with worst outcome for DLBCL patients treated with axicabtagene ciloleucel (axi-cel) [159]. The association with checkpoint inhibitors can represent a safe strategy to restore microenvironment activity and optimize CAR-T cell effectiveness, even if there are conflicting reports concerning CAR-T cell expansion following exposure to PD1 inhibitors [160,161,162]. A trial adding PD-1 inhibitor pembrolizumab to CAR-T cell therapy drugs lisocabtagene maraleucel (liso-cel) or axi-cel is actively recruiting (NCT05934448).

Several ongoing trials are investigating the addition of drugs to anti-CD19 CAR-T cell treatments. Candidates like mosunetuzumab and polatuzumab (NCT05260957), selinexor (NCT05322330), tazemetostat (NCT05934838), glofitamab (NCT04889716), and obinotuzumab (NCT04889716) are being explored. Additionally, research is underway to assess the combination of ASCT with anti-CD19 CAR-T cell therapy (NCT05239676).

In the following sections, we discuss the main results of CAR-T treatments according to the type of NHL.

### 9.1. Indolent Lymphomas

Tisagenlecleucel (tisa-cel) and axi-cel have recently received approval from both the FDA and EMA for adults with R/R FL after ≥2 prior lines of therapy after the publication in 2022 of the results of the ELARA and ZUMA-5 trials, which reported high CR rates (69% and 76%, respectively) [163,164]. In the setting of indolent lymphomas, longer follow-ups are needed to evaluate the curative potential of these agents.

At ASH 2023, the three-year follow-up from the phase II ELARA trial was reported, confirming the high ORR and CR rates (86% and 68%, respectively) in the evaluable 94 patients. Median PFS was 37 months, with a 3-year PFS rate of 53% in all patients and 69% in patients who achieved a CR as best response. The 3-year OS rate was 82%, and the probability of starting a new treatment was 35%. Interestingly, high baseline levels of circulating CD8+ naive T cells were associated with prolonged PFS and DoR, suggesting that probably administrating this treatment in earlier phases of the disease (median number of four previous lines in the ELARA trial) could improve the outcome [165].

Following this direction, in the phase II TRANSCEND FL trial, high-risk (high FLIPI and/or POD24) R/R FL patients (*n* = 23) were treated with the CAR-T product liso-cel already in the second-line setting. At a median follow-up of 18 months the ORR and CR rate were both 96%, with 12-month DoR and PFS rate of 89.8% and 91.3%, respectively. Despite the lack of adequate follow-up to draw any final conclusion, these preliminary data support the efficacy of CAR-T in high-risk R/R FL patients, suggesting a potential role in clinical settings, those of POD24, which is today an unmet medical need [166].

### 9.2. CLL

In the phase I/II TRANSCEND CLL 004, R/R CLL patients were treated with a single infusion of the CAR-T product liso-cel. A total of 88 patients were evaluable for efficacy, with 50/88 patients in failure after receiving both a cBTKi and a BCL2i. In the entire population, 83% had high-risk cytogenetics (17p deletion, 42%; *TP53* mutation, 47%; unmuted immunoglobulin heavy-chain variable gene, 47% and; ≥3 chromosomal aberrations, 61%). In the entire population, ORR was 47% and CR rate was 19%, while for double refractory patients ORR was 44% and CR rate was 20%. At a follow-up of 23.5 months, the median PFS was 17.9 months for the entire population and 11.9 for the double refractory cohort with a median DoR of CR which was NR in the two groups. The rate of any-grade CRS was 85% (grade 3, 8%; no grade, four-fifths) and neurological events was 45% (grade 3, 18%; grade 4, 1%; no grade, 5). Liso-cel showed high efficacy in this study enrolling heavily pretreated patients (five median prior lines of treatment) and the long DoR of CR is particularly remarkable, suggesting opportunities for improvement through an earlier referral to CAR-T [167].

### 9.3. DLBCL

The phase II ZUMA-12 trial proposed axi-cel treatment to patients with DLBCL and an IPI score ≥ 3 DLBCL or a double/triple-hit histology, who were not in CR after two cycles of immunochemotherapy containing an anthracycline and an anti-CD20 compound. A recent update on 37 eligible patients, at a median follow-up of 40.9 months, showed an ORR and a CRR of 92% and 86%, respectively. The 3-year PFS and 3-year OS rates were 75% and 81%, respectively. During follow-up, eight patients died, mainly due to progressive disease (*n* = 5) [168]. These results highlight the potential benefit of axi-cel as a frontline therapy for high-risk lymphoma patients and the potentially higher efficacy of CAR-T as a first-line treatment. Phase III trials comparing axi-cel with standard immunochemotherapies are needed. 

### 9.4. PMBCL

CAR-T therapy may be a real game changer for R/R PMBCL, as it also leads to cure in chemorefractory patients. In the TRANSCEND trial, 14 R/R PMBCL patients received a treatment with lisocabtagene maraleucel (liso-cel), showing an ORR of 79% and a CR rate of 50% [169]. Moreover, a real-world experience of axi-cel was recently published, reporting impressive results with an ORR of 76% and a CR rate of 67% in an intention-to-treat population [169]. The 24-month PFS rate was 64% and the adverse event profile was manageable [170]. Interestingly, patients receiving anti-PD1 therapy after CAR-T cell therapy showed better results, suggesting that this combined strategy could improve the outcome in this specific setting [170].

### 9.5. PCNSL

CAR-T therapy constitutes a very promising approach in PCNSL regarding available data, although no consensus has been established yet. Initially, CAR-T clinical trials have mainly excluded patients with CNS involvement because of the supposed risk of neurotoxicity, except the TRANSCEND trial [169]. Indeed, seven B-cell lymphoma patients with CNS disease were included in the study and out of the six evaluable patients, 50% achieved a CR with only two patients having developed a grade 3 immune effector cell-associated neurotoxicity syndrome (ICANS) [169]. Based on those encouraging results and many more reports of CAR-T cell therapy in secondary CNS lymphomas, prospective trials were conducted. Recently, a phase I-II trial (NCT02445248) reported the outcome of 14 R/R PCNSL patients treated with tisa-cel, showing an ORR of 58% with 50% CRs and only one patient developing a reversible grade 3 ICANS [171]. Numerous prospective trials are currently ongoing trying to consolidate those highly encouraging results while some innovative combinations are under investigation as well, such as PD1 inhibitors and CAR-T therapies (Phase I CAROUSEL trial—NCT04443829).

### 9.6. HL

While CAR-T-cell development for HL is in its early stages, autologous anti-CD30 CAR-T cells are showing promising results [172]. Preliminary reports indicate good efficacy outcomes without safety concerns, with ongoing trials such as allogeneic CD30.CAR-EBVSTs (NCT04288726) and auto anti-CD30 CAR-T cells in combination with PD1 inhibitors (NCT04134325).

### 9.7. T-Cell Malignancies

Advancements in CAR-T therapy for T lymphomas are challenged by intrinsic issues related to T cells in these pathologies. Targeting CD7, CD5, and CD56 seems useful to overcome hurdles such as fratricide effect and tumoral cell contamination of CAR product. Innovations like Protein Blocker (PEBL) and CRISPR/Cas9 gene deletion, to avoid fratricide effect and pharmacologic inhibition with ibrutinib and dasatinib, are enhancing the effectiveness of anti-CD7 and anti-CD5 CAR-T cells [173,174]. Ongoing trials, including those investigating donor-derived anti-CD5 CAR-T cells (NCT05487495, NCT03081910) and anti-CD56 CAR-T cells (NCT05941156), are enriching the landscape of cellular therapies for T-cell malignancies.

A summary of the treatments discussed in this paragraph and its subsections is displayed in Table 8.

## 10. Conclusions

The landscape of lymphoproliferative disorder treatment is evolving rapidly, driven by an expanding armamentarium of targeted therapies, as summarized in Table 9. Many efforts have been made and are currently ongoing to incorporate immunotherapy in clinical practice, delineating its role across various disease phases and settings. With the advent of therapies such as BV and checkpoint inhibitors, there is potential to achieve improved outcomes while mitigating long-term toxicities associated with traditional chemotherapy. Cellular therapies, particularly CAR-T, emerge as a potential revolution for the R/R of several types of NHL, considered incurable until a few years ago. Nonetheless, these advancements call for further investigations, especially in optimizing combination strategies, managing resistance mechanisms, and refining administration timing. The promising horizon in treating lymphoproliferative disorders is undeniably marked by the innovative therapeutic approaches reviewed herein, signifying a shift towards a future where treatment is not only effective but also patient-centric and toxicity-spared.

## Figures and Tables

**Table 1:** A summary of the treatments discussed in the section “Early-phase/basket trials”. NR = not reached.

Treatment	Trial Phase	Target	ORR	CR	mDoR
Golcadomide	I/II	Ikaros/Aiolos	50%	13%	17.4 w
LP-168	I	BTK	65% (MCL: 77%, DLBCL: 70%, MZL: 73%)	MCL: 39%, DLBCL: 40%, MZL: 9%	/
Emavusertib	I	IRAK4	38%	31%	/
HZ-H08905	I	CK1ε/PI3Kδ	60%	17%	NR
ZD8586	I	LYN/BTK	69%	/	/
Zilovertamab	I	ROR1	MCL: 53%, DLBCL: 29%, RT: 57%	/	/
AR160	I	CD20	85%	14%	/

**Table 2:** A summary of the treatments discussed in the section “Indolent NHL”.

Treatment	Trial Phase	Setting	Target	ORR	CR	DoR	PFS
Acalabrutinib− Rituximab− Lenalidomide	II	First-line FL	BTK/CD20/ IMiD	100%	92%	/	
Mosunetuzumab ± Lenalidomide	II	First-line FL	CD20xCD3/ IMiD	96% (M) 89% (M + L)	81% (M) 82% (M + L)	/	/
Epcoritamab	II	R/R FL	CD20xCD3	90%	50%	/	/
Glofitamab	II	R/R FL	CD20xCD3	81%	70%	/	/
Odronextamab	II	R/R FL	CD20xCD3	91%	72%	/	/
Zanubrutinib− Obinutuzumab	II	R/R FL	BTK/CD20	69%	39%	18 m: 69%	mPFS: 28 m
Loncastuximab tesirine	II	R/R FL	CD19	95%	67%	/	/
Zanubrutinib− Venetoclax− Obinutuzumab	II	R/R MCL	BTK/BCL2/ CD20	96%	88%	/	2y: 72%
Ibrutinib− Venetoclax	III	R/R MCL	BTK/BCL2	82%	54%	mDoR: 42 m	mPFS: 32 m
Pirtobrutinib	I/II	R/R MCL	BTK			2y: 86% (cBTKn) vs. 90% (cBTKexp)	

**Table 3:** A summary of the treatments discussed in the section “Chronic lymphocytic leukemia (CLL)”.

Treatment	Trial Phase	Setting	Target	ORR	CR	DoR	PFS
Ibrutinib− Venetoclax	III	First line	BTK/BCL2	95%	92%	/	3y: 97%
Obinutuzumab− Venetoclax ± Ibrutinib	III	First line	CD20/BCL2/ BTK	96% (O + V) vs. 94% (O + V + I)	57% (O + V) vs. 62% (O + V + I)	/	4y: 82% (O + V) vs. 86% (O + V + I)
Venetoclax− Rituximab	III	R/R retreatment cohort	BCL2/CD20	72%	56%	/	mPFS: 23 m
Ibrutinib− Venetoclax	II	R/R Retreatment cohort	BTK/BCL2	86%	5%	/	/
Pirtobrutinib	I/II	R/R	BTK	83% (cBTKn) vs. 80% (cBTKexp)	/	mDoR: 25 m (cBTKn) vs. 15 m (cBTKexp)	mPFS: 23 m (cBTKn) vs. 16 m (cBTKexp)
Epcoritamab− Venetoclax	I/II	R/R	CD20xCD3/ BCL2	82%	33%	9 m: 83%	/

**Table 4:** A summary of the treatments discussed in the section “Diffuse large B-cell lymphoma (DLBCL) and primary mediastinal B-cell lymphoma (PMBCL)”.

Treatment	Trial Phase	Setting	Target	ORR	CR	PFS	OS
Polatuzumab vedotin− Rituximab−CHP	III	First-line DLBCL	CD79b	86%	78%	2y: 77%	2y: 89%
Rituximab− Lenalidomide− Ibrutinib− CHOP/EPOCH	II	First-line DLBCL	CD20/IMiD/ BTK	100%	94%	31m: 91%	31m: 97%
Lenalidomide− Tafasitamab− Rituximab− Acalabrutinib	II	First-line DLBCL	IMiD/CD19/ CD20/BTK	100%	64%	/	/
Polatuzumab vedotin− Rituximab− Bendamustine	III	R/R DLBCL	CD79b/CD20	/	43%	mPFS: 9m	mOS: 12m
Tafasitamab− Lenalidomide	II	R/R DLBCL	CD79b/IMiD	60%	43%	mPFS: 11m	mOS: 33m
Epcoritamab	II	R/R DLBCL	CD20xCD3	63%	39%	mPFS: 4m	mOS: NR
Glofitamab	II	R/R DLBCL	CD20xCD3	52%	39%	mPFS: 5m	1y: 50%
Pembrolizumab	II	R/R PMBCL	PD-L1	41%	21%	4y: 33%	4y: 45%
Brentuximab Vedotin	II	R/R PMBCL	CD30	70%	43%	mPFS: NR	mOS: NR

**Table 5:** A summary of the treatments discussed in the section “Primary central nervous system lymphoma (PCNSL)”.

Treatment	Trial Phase	Setting	Target	ORR	CR	PFS	OS
Lenalidomide− Rituximab− Methotrexate− Temozolomide	II	First line	IMiD/CD20	92%	79%	2y: 62%	2y: 67%
Ibrutinib	II	R/R	BTK	59%	19%	mPFS: 5m	mOS: 20m
Lenalidomide− Rituximab	II	R/R	IMiD/CD20	67%	/	mPFS: 8m	mOS: 18m
Pomalidomide− Dexamethasone	I	R/R	IMiD	48%	/	mPFS: 6m	/

**Table 6:** A summary of the treatments discussed in the section “Hodgkin lymphoma (HL)”.

Treatment	Trial Phase	Setting	Target	ORR	CR	PFS	OS
Brentuximab vedotin− AVD	III	First-line advanced stage	CD30	/	/	6y: 82%	6y: 94%
Brentuximab vedotin	II	First-line frail	CD30	84%	26%	mPFS: 7m	mOS: 20m
Brentuximab vedotin− AVD (sequential)	II	First-line Frail	CD30	82%	36%	2y: 84%	2y: 93%
Nivolumab− AVD	III	First-line advanced	PD-L1	/	/	1y: 94%	/
Brentuximab vedotin− Nivolumab	II	First-line Frail	CD30/ PD-L1	61%	48%	/	/
Brentuximab vedotin−ICE	I/II	R/R	CD30	/	62%	3y: 64%	3y: 100%
Brentuximab vedotin−IGEV	I/II	R/R	CD30	96%	71%	/	/
Brentuximab vedotin−ESHAP	I/II	R/R	CD30	91%	70%	30m: 71%	30m: 91%
Brentuximab vedotin−DHAP	I/II	R/R	CD30	91%	81%	2y: 74%	2y: 95%
Pembrolizumab− ICE	II	R/R	PD-L1	97%	87%	2y: 87%	2y: 95%
Pembrolizumab− GVD	II	R/R	PD-L1	100%	95%	14m: 100%	14m: 100%
Brentuximab vedotin− Nivolumab	II	R/R	CD30/ PD-L1	85%	67%	3y: 77%	3y: 93%
Brentuximab vedotin− Pembrolizumab	II	R/R	CD30/ PD-L1	/	80%	/	/

**Table 7:** A summary of the treatments discussed in the section “Peripheral T-cell lymphoma, not otherwise specified (PTCL-NOS)”.

Treatment	Trial Phase	Setting	Target	ORR	CR	PFS	OS
Duvelisib	II	R/R	PI3K	49%	34%	mPFS: 4m	/
Linperlisib	Ib	R/R	PI3K	60%	35%	mPFS: 10m	mOS: NR
Copanlisib	II	R/R	PI3K	21%	14%	/	/
Bortezomib	II	R/R	Proteasome	67%	17%	/	/
Ixazomib	II	R/R	Proteasome	14%	14%	/	/
Valemetostat	I	R/R	EZH1/2	55%	31%	mPFS: 7.7m	/
Romidepsin− pralatrexate	I	R/R	HDAC	71%	/	/	/
Romidepsin− Duvelisib	II	R/R	HDAC/ PI3K	47%	29%	/	/
Romidepsin− lenalidomide	II	R/R	HDAC/ IMiD	65%	26%	2y: 32%	2y: 50%
Romidepsin− azacitidine	II	R/R	HDAC/ DNA methylation	80%	67%	/	/
AZD4573	II	R/R	CDK9	25%	25%	/	/
HH2853	Ib	R/R	EZH1/2	61%	39%	3m: 74%	6m: 92%
Golidocitinib	II	R/R	JAK1	39%	33%	NR	/

**Table 8:** A summary of the treatments discussed in the section “Cellular therapy in lymphoproliferative disorders”.

Treatment	Trial Phase	Setting	Target	ORR	CR	PFS	OS
Tisa-cel	III	R/R FL	CD19	69%	76%	mPFS: 37m	3y: 82%
Liso-cel	II	R/R FL	CD19	96%	96%	12m: 90%	12m: 91%
Liso-cel	I/II	R/R CLL	CD19	47%	19%	mPFS: 18m	/
Axi-cel	II	Refractory (two cycles of chemotherapy) DLBCL	CD19	92%	86%	3y: 75%	3y: 81%
Liso-cel	II	R/R PMBCL	CD19	79%	50%	/	/
Axi-cel	II	R/R PMBCL	CD19	76%	67%	2y: 64%	/
Tisa-cel	I/II	R/R PCNSL	CD19	58%	50%	/	/

**Table 9:** A summary of the key messages for each section.

**Early phase**	New molecular pathways (Ikaros/Aiolos, IRAK4, and ROR1) and new delivery systems (nanotechnology) are currently being evaluated in phase I/II trials for various types of NHL.
**FL/MCL**	Bispecific antibodies showed important activity in R/R settings and are now moving to first line in FL. In MCL, new chemo-free regimens combining a cBTKi and a BCL2i also showed promising activity in patients with TP53 aberrations.
**CLL**	Fixed-duration schemes and continuous treatments with cBTKi are available options for CLL. New randomized trials directly comparing these different approaches and/or evaluating MRD-driven strategies are ongoing.
**DLBCL/** **PMBCL**	Targeted therapies such as polatuzumab vedotin and bispecific antibodies are changing the management of DLBCL. Chemo-free regimens based on checkpoint inhibitors seem promising in PMBCL.
**PCNSL**	Targeted therapies like ibrutinib and immunomodulation with lenalidomide emerge for PCNSL, with studies showing encouraging initial responses.
**HL**	The use of BV and checkpoint inhibitors improves outcomes in HL, with new combinations and sequential approaches demonstrating efficacy and tolerability.
**PTCL-NOS**	New agents such as azacitidine, romidepsin, and JAK inhibitors showed promising outcomes for PTCL-NOS in phase I/II studies.
**Cellular therapy**	CAR-T cell treatment is emerging as a revolution for R/R NHL, with studies showing high efficacy and a curative potential. New approaches, like the development of CAR-NK cells or allogenic CAR-T cells, are currently under investigation.

## Data Availability

Not applicable.
